# Interactions of *miR-34b/c* and *TP53* Polymorphisms on the Risk of Intracranial Aneurysm

**DOI:** 10.1155/2012/567586

**Published:** 2012-07-11

**Authors:** Lijuan Li, Xiutian Sima, Peng Bai, Lushun Zhang, Hong Sun, Weibo Liang, Jianxing Liu, Lin Zhang, Linbo Gao

**Affiliations:** ^1^Laboratory of Molecular and Translational Medicine, West China Institute of Women and Children's Health, West China Second University Hospital, Sichuan University, Chengdu 610041, China; ^2^Department of Forensic Biology, West China School of Preclinical and Forensic Medicine, Sichuan University, Chengdu 610041, China; ^3^Department of Neurosurgery, West China Hospital, Sichuan University, Chengdu 610041, China; ^4^Department of Forensic Medicine, Kunming Medical University, Kunming 650031, China; ^5^Key Laboratory of Obstetric & Gynecologic and Pediatric Diseases and Birth Defects of Ministry of Education, Chengdu, Sichuan 610041, China

## Abstract

Several lines of evidence indicate that inflammatory processes play a key role in the happening and development of intracranial aneurysm (IA). Recently, polymorphisms in the *TP53* gene were shown to be associated with inflammation and inflammatory disease. The aim of this study was to investigate the interactions of *miR-34b/c* and *TP53* Arg72-Pro polymorphisms on the risk of IA in a Chinese population. A total of 590 individuals (including 164 patients with IA and 426 controls) were involved in this study. The polymorphisms (i.e., *miR-34b/c* rs4938723 and *TP53* Arg72-Pro) were genotyped by polymerase chain reaction-restriction fragment length polymorphism assay and DNA sequencing. We found that the CC genotype of *miR-34b/c* rs4938723 was significantly associated with a decreased risk of IA compared with the TT genotype. Moreover, a significant gene interaction of the carriers with the combined genotypes of *miR-34b/c* rs4938723CC and *TP53* Arg72Pro CG/CC/GG had a decreased risk of IA, compared with those carrying *miR-34b/c* rs4938723CT/TT+*TP53* Arg72Pro GG/CG/CC combined genotypes. These findings suggest that the *miR-34b/c* rs4938723CC and *TP53* Arg72-Pro polymorphisms may be involved in the susceptibility to IA.

## 1. Introduction

Intracranial aneurysm (IA) is a common disease with a high prevalence ranging from 1 to 5 percent in large autopsy studies [1]. Rupture of IAs causes approximately 75% of all subarachnoid hemorrhage (SAH) cases and most ruptured IAs present with SAH [2–7]. SAH remains a critical condition, with only 25% of victims living independently [8]. Inflammation was firstly suggested to occur in IAs by Virchow in 1847 [9], and further evidence came from the 1930s when Maass [10, 11] described round cell infiltration, most likely lymphocytes that have been regularly detected in immunohistochemical studies of the IA wall [12–14] are associated with IA rupture [15, 16]. In experimental IA in rodents, macrophage infiltration goes after IA formation and endothelial dysfunction [17, 18]. The role of inflammation in the formation and progression of aneurysm has not been well investigated, but there was considerable circumstantial evidence linking inflammation to IA [12, 19–21]. 

Both environmental and genetic factors are involved in the etiology of IA [22, 23]. Several studies have revealed candidate genes in different populations [24–34]. Indeed, there is a three- to fivefold increased risk for first-degree relatives of affected individuals, compared with the general population [35, 36]. The *TP53* gene has an important function in cell cycle control, apoptosis, and maintenance of DNA integrity [37–39]. The importance of p53 in cell cycle regulation and DNA integrity is such that it has been called the “guardian of the genome” [40]. *TP53*, located on chromosome 17p13 [41, 42] is 19 kb in size and consists of 11 exons resulting in a transcript of 2629 bp and a protein of 393 amino acids [43]. Codon 72 (Arg72Pro) in exon 4 of the *TP53* gene is a frequent functional SNP that leads to a methionine proline conversion [44, 45]. The *TP53* Arg72Pro SNP results in a change in its protein structure reflected by its altered electrophoretic mobility [46], and this SNP exists only in humans [45]. More importantly, the Arg72Pro polymorphism of *TP53* was reported to influence the p53-mediated inflammatory response [47].

It is well known that p53 can regulate the expression of miRNAs, especially the *miR-34* family members, which compose 3 mature miRNAs that are encoded by 2 different pri-miRNAs [48–54]. The promoter regions of both transcripts contain p53-binding sites [49]. A potentially functional common SNP rs4938723 (T > C) has been found in the promoter region of *miR-34b/c, *which may contribute to the susceptibility of HCC in a Chinese population [21].

In this study, we hypothesized that SNPs rs4938723 (T > C) in the promoter region of *miR-34b/c* and *TP53* Arg72-Pro were associated with the risk of IA. To test this hypothesis, we genotyped the 2 SNPs in a case-control study of 164 IA patients and 426 healthy controls in a Chinese population.

## 2. Subjects and Methods

### 2.1. Study Populations

The study was performed with the approval of the hospital ethics committee, and written informed consent was obtained from all subjects participating in this study. The case-control study population contained 590 unrelated Chinese Han individuals including 164 patients (60 males and 104 females, mean age: 53.1 (±13.1)) with IA and 426 healthy controls (205 males and 221 females, mean age: 51.3 (±8.9)) living in Sichuan province of southwest China. Patients were recruited from the West China Hospital, Sichuan University from January 2008 to September 2009 who were newly diagnosed when they came for emergency because of SAH caused by the rupture of IA or just had general clinical symptoms such as headache or dizziness and diagnosed by DSA (digital subtraction angiography). The control group consisted of 426 healthy volunteers from a routine health survey in the same hospital during the same time as the patients. Subjects with any disease in nervous system or other serious illness were intentionally excluded. There was no significant difference between patients and control subjects in age distribution.

### 2.2. Genotyping

Genomic DNA was extracted from 200 *μ*L EDTA-anticoagulated peripheral blood using a commercial extraction kit (Bioteke Corporation, Beijing, China) according to the instruction manual. We used a polymerase chain reaction-restriction fragment length polymorphism assay to detect the genotype of the two SNPs (i.e., *miR-34b/c* rs4938723 and *TP-53* Arg72-Pro). Primer sequences, reaction conditions, restriction enzymes (New England BioLabs Inc; Beverly, MA, USA) used, and length of resulting polymerase chain reaction products have been described previously [21]. Restriction fragments were distinguished on 6% polyacrylamide gel and stained with 1.0 g/L argent nitrate to determine the genotypes. The PCR products of the two SNPs with different genotypes were randomly selected to be confirmed by DNA sequencing, and the results were 100% consistent.

### 2.3. Statistical Analysis

In this retrospective study, demographic and clinical data of both groups were compared by the chi-square test and *t* test. Genotype and allele frequencies of *miR-34b/c* rs4938723 and *TP53* Arg72Pro were obtained using Modified-Powerstates standard edition software. Hardy-Weinberg equilibrium was tested with chi-square test to compare the observed genotype frequencies among the subjects with the expected genotype frequencies. Genotype and allele frequencies of the two SNPs were compared between IA cases and controls using the chi-square tests. Odds ratios (ORs) and 95% confidence intervals (CIs) were used to assess the relative risk conferred by a particular allele, genotype, or the combined genotypes of *miR-34b/c* rs4938723 and *TP53* Arg72Pro. Statistical significance was set at the *P* < 0.05 level. All the data were analyzed using the SPSS for windows software package version 13.0 (SPSS Inc., Chicago, IL).

## 3. Results

The two SNPs of *miR-34b/c* rs4938723 and *TP53* Arg72Pro were successfully genotyped for 164 patients with IA and 426 controls. The clinical characteristics including sex, age of both groups, and the number of aneurysms in the cases enrolled in our study are shown in Table 1. The mean age of all subjects was almost identical. There were more females among the cases (63.4%) than those in the controls (51.9%). The genotype and allele frequencies distribution of both polymorphisms in the control group met the requirements of the Hardy-Weinberg equilibrium. The genotype and allele frequencies of the two SNPs and the combined genotypes frequencies of *miR-34b/c* rs4938723 and *TP53* Arg72Pro are summarized in Tables 2, 3 and 4. The CC genotype of *miR-34b/c* rs4938723 was significantly associated with a decreased risk of IA, compared with the TT genotype (OR = 0.28, 95% CI: 0.11–0.73, *P* = 0.006). However, no association between *TP53* Arg72Pro and the risk of IA was observed for genotypic or allelic association analysis. We also examined the combined effects of *miR-34b/c* rs4938723 and *TP53* Arg72Pro variants on IA risk. As shown in Table 3, the carriers with the combined genotypes of rs4938723CC and *TP53* Arg72Pro CG/CC/GG had a 0.27-fold decreased risk of IA (OR = 0.27, 95% CI: 0.11–0.70, *P* = 0.004), compared with those carrying rs4938723CT/TT + *TP53* Arg72Pro CG/CC/GG combined genotypes, while no other association between combined effects of *miR-34b/c* rs4938723 and *TP53* Arg72Pro variants on IA risk was found. When stratification analysis was done by gender, no association was found (Tables 5 and 6). No significant association between the two SNPs and the number of aneurysms was observed (Table 7).

## 4. Discussion

In this study, we investigated the association between two SNPs (i.e., *miR-34b/c* rs4938723 and *TP53* Arg72Pro) and the risk of IA in a Chinese population. We found that the CC genotype of *miR-34b/c* rs4938723 was significantly associated with a decreased risk of IA, compared with the TT genotype. Moreover, a gene interaction of the carriers with the combined genotypes of *miR-34b/c* rs4938723CC and *TP53* Arg72Pro CG/CC/GG had a decreased risk of IA, compared with those carrying *miR-34b/c* rs4938723 CT/TT + *TP53 *Arg72Pro CG/CC/GG combined genotypes. However, no difference was found when stratification analysis was done by gender for both *miR-34b/c* rs4938723CC and *TP53* Arg72Pro.

There are two *miR-34* loci in vertebrate genomes, one encoding *miR-34a* and the other generating both *miR-34b* and* miR-34c*. Both genes show little conservation even among closely related species, except in the miRNA-encoding sequences and in short promoter proximal regions that each contains a consensus p53-binding site [48–54]. Polymorphisms in miRNA genes may alter miRNA processing by changing the stem-loop structure. Although this is not an active processing regulation mechanism, it is evident that SNPs do alter the processing efficiency [55]. Several candidate genes have been analyzed in order to investigate the possible impact of inflammation-associated SNP on the development of IA [56–62]. When it comes to *TP53,* the inflammatory microenvironment both activates the p53 network and inactivates the tumor suppressor activity by mutation of the p53 gene [63–65]. Another link between p53 and inflammation is suggested by studies demonstrating the presence of *TP53* mutations in areas of rheumatoid arthritis (RA) synovial tissues [66–69]. Frank et al. [47] reported that the Arg72Pro polymorphism of *TP53* influences the p53-mediated inflammatory response. The role of p53 in innate immunity and the inflammatory response is now well established [70–72] and, importantly, is evolutionarily conserved [73]. Also a rat model experiment indicated that inflammation modulates miRNA expression *in vivo* and the alteration of *miR-34b/c* under an inflammatory microenvironment can be influenced by p53 [74].

Although the molecular mechanisms by which *miRNA *and* p53* gene polymorphisms are associated with IA remain unclear, additional functional studies would provide valuable characterization of the molecular mechanisms by which *miRNA *and* TP53*  are involved in susceptibility to IA. However, our study provides evidence that* miRNA *and* TP53 *polymorphisms may play an important role in individuals' susceptibility to IA.

There were several limitations in our study. Firstly, detailed lifestyle that may be involved in the occurrence and development of IA was not available. Secondly, the number of subjects in the study was so small which might not be a good representative of the general population. Further studies, therefore, still need to be done.

In conclusion, we found that the CC genotype of *miR-34b/c* rs4938723 was significantly associated with a decreased risk of IA, and a significant gene interaction of *miR-34b/c* rs4938723CT/TT and *TP53* Arg72Pro CG/CC/GG was evident on the risk of IA. These findings suggest that *miR-34b/c* rs4938723 and* TP53* Arg72Pro play a role in the formation, process, or rupture of IA. Nevertheless, our findings need to be replicated in larger, preferably multiethic population-based studies.

## Figures and Tables

**Table 1:** Demographics of the patients with intracranial aneurysm and controls.

Variables	Controls *n* = 426 (%)	Patients with intracranial aneurysm *n* = 164 (%)
Age (y)	51.3 (±8.9)	53.1 (±13.1)
Sex		
Male	205 (48.1)	60 (36.6)
Female	221 (51.9)	104 (63.4)
Number of aneurysms		
1	—	142
>1	—	22

**Table 2:** Genotype frequencies of *miR-34 *rs4938723 and *TP-53* Arg72Pro between patients with intracranial aneurysm and controls.

Polymorphisms	Controls *n* = 426 (%)	Patients *n* = 164 (%)	OR (95% CI)	*P* value
rs4938723				
TT	188 (44.1)	77 (47.0)	1.0 (ref)	
CT	194 (45.6)	82 (50.0)	1.03 (0.71–1.49)	0.87
CC	44 (10.3)	5 (3.0)	0.28 (0.11–0.73)	0.006
*TP-53*				
GG	145 (34.0)	60 (36.6)	1.0 (ref)	
CG	223 (52.4)	77 (46.9)	0.83 (0.56–1.24)	0.37
CC	58 (13.6)	27 (16.5)	1.13 (0.65–1.94)	0.67

OR: odds ratio.

CI: confidence interval.

Ref: reference.

**Table 3:** The combined genotypes frequencies of *miR-34 *rs4938723 and *TP-53* Arg72Pro between patients with intracranial aneurysm and controls.

Polymorphisms	Controls *n* (%)	Patients *n* (%)	OR (95% CI)	*P* value
rs4938723CT/TT + TP53CG/CC	255 (66.8)	100 (62.9)	1.0 (ref)	
rs4938723CT/TT + TP53GG	127 (33.2)	59 (37.1)	1.19 (0.81–1.74)	0.39
rs4938723CT/TT + *TP-53 CG/CC/GG *	382 (89.7)	159 (97.0)	1.0 (ref)	
rs4938723CC + *TP-53 CG/CC/GG *	44 (10.3)	5 (3.0)	0.27 (0.11–0.70)	0.004

OR: odds ratio.

CI: confidence interval.

Ref: reference.

**Table 4:** Allele frequencies of *miR-34 *rs4938723 and *TP-53* Arg72Pro between patients with intracranial aneurysm and controls.

Alleles	Controls *n* = 426 (%)	Patients *n* = 164 (%)	OR (95% CI)	*P* value
rs4938723				
T	570 (66.9)	236 (72.0)	1.0 (ref)	
C	282 (33.1)	92 (28.0)	0.79 (0.60–1.04)	0.10
*TP-53*				
G	513 (60.2)	197 (60.1)	1.0 (ref)	
C	339 (39.8)	131 (39.9)	1.01 (0.78–1.31)	0.96

OR: odds ratio.

CI: confidence interval.

Ref: reference.

**Table 5:** Genotype frequencies of *miR-34 *rs4938723 and *TP-53* Arg72Pro in female subjects.

Polymorphisms	Controls *n* = 221 (%)	Patients *n* = 104 (%)	OR (95% CI)	*P* value
rs4938723				
TT	96 (43.4)	42 (40.4)	1.0 (ref)	
CT	103 (46.6)	59 (56.7)	1.31 (0.81–2.12)	0.27
CC/CT	125 (56.6)	62 (59.6)	1.13 (0.71–1.82)	0.60
*TP-53*				
GG	74 (33.5)	39 (37.5)	1.0 (ref)	
CG	117 (52.9)	47 (45.2)	0.76 (0.46–1.28)	0.30
CC	30 (13.6)	18 (17.3)	1.14 (0.57–2.30)	0.72

OR: odds ratio.

CI: confidence interval.

Ref: reference.

**Table 6:** Genotype frequencies of *miR-34 *rs4938723 and *TP-53* Arg72Pro in male subjects.

Polymorphisms	Controls *n* = 205 (%)	Patients *n* = 60 (%)	OR (95% CI)	*P* value
rs4938723				
TT	92 (44.9)	35 (58.3)	1.0 (ref)	
CT	91 (44.4)	23 (38.3)	0.66 (0.36–1.21)	0.18
CC/CT	113 (55.1)	25 (41.7)	0.58 (0.33–1.04)	0.07
*TP-53*				
GG	71 (34.6)	21 (35.0)	1.0 (ref)	
CG	106 (51.7)	30 (50.0)	0.96 (0.51–1.80)	0.89
CC	28 (13.7)	9 (15.0)	1.09 (0.44–2.66)	0.86

OR: odds ratio.

CI: confidence interval.

Ref: reference.

**Table 7:** Stratified analysis by the number of aneurysms.

Polymorphisms	The number of aneurysms		OR (95% CI)	*P* value
	1	>1		
	*n* = 142	*n* = 22		
rs4938723				
TT	64 (45.1)	13 (59.1)	1.0 (ref)	
CT/CC	78 (54.9)	9 (40.9)	0.57 (0.23–1.41)	0.22
*TP-53*				
GG	53 (37.3)	7 (31.8)	1.0 (ref)	
CG/CC	89 (62.7)	15 (68.2)	1.28 (0.49–3.33)	0.62

OR: odds ratio.

CI: confidence interval.

Ref: reference.
